# Data on gender-equitable healthcare accessibility in Northern Nigeria

**DOI:** 10.1016/j.dib.2023.109979

**Published:** 2023-12-18

**Authors:** Chika Yinka-Banjo, Mary Akinyemi, Olasupo Ajayi, David Tresner-Kirsch

**Affiliations:** a Department of Computer Science, University of Lagos, Lagos 101017, Nigeria; b AiRoL Lab, University of Lagos, Lagos 101017, Nigeria; c Department of Mathematics & Statistics, Austin Peay State University, TN 37040, USA; d Department of Computer Science, Brandeis University, 415 South Street, Waltham, MA 02453, USA; e Nivi Inc., 40 Tall Pine Drive, Sudbury, MA 01776, USA

**Keywords:** Healthcare, Gender-equity, Regional dataset, Nigeria

## Abstract

Gender equity, particularly in healthcare, has been gaining increasing attention in recent years. The goal is to ensure that everyone has equal access to quality healthcare services irrespective of age, gender, or socio-economic status. However, most countries in sub-Saharan Africa struggle to meet this goal, due to several challenges, including poverty, poor infrastructure, and gender-bias. Using Nigeria as a case-study, it is common knowledge that gender inequality and discrimination is predominant in the northern region of the country. This work sought to gather data to assess the level of healthcare accessibility from a gender-based perspective in northern Nigeria. Data were sourced anonymously from residents in about 500 locations across the northern region of Nigeria, using WhatsApp-based questionnaires, in two phases and two languages - English and Hausa. About 4700 participants took part in the survey and each had to answer 43 questions, split into demographic, socio-economic, wellness check, and diversity, equity, and inclusion (DEI) in health care services obtained.

Specifications Table

**Table:** 

Subject	Health and medical sciences
Specific subject area	Healthcare accessibility, Gender-equity.
Data format	Raw
Type of data	Table
Data collection	Data were collected from patients of various healthcare centres through questionnaires administered *via* a mobile application [1], and in two languages - English and Hausa language. In total, four datasets were curated; the first being the responses from an initial pilot study, the second containing English language responses, the third Hausa language responses, and a fourth containing English translations of Hausa words in the Hausa dataset. All datasets have 43 variables, except the fourth which has 51 variables (43 plus 8 additional variables containing English translations of Hausa words from the Hausa dataset).
Data source location	Data on healthcare accessibility were collected from about 500 towns and villages across northern Nigeria. The Latitude and Longitude of the collection region are: Upper left corner = 13°01′58.368"N and 5°21′56.952"E Upper right corner = 11°49’54.264”N and 13°10’0.551”E Lower left corner = 9°16’4.404”N and 5°20’43.943”E Lower right corner = 9°10’25.356”N and 12°24’54.467”E
Data accessibility	Repository name: Mendeley Data. Title: Data on Gender-Equitable Healthcare Accessibility in Northern Nigeria DOI: 10.17632/8gbywtd7bv.2 Direct URL to data: https://data.mendeley.com/datasets/8gbywtd7bv/2

## 1 Value of the Data

• This data is useful for assessing gender-based healthcare accessibility in northern Nigeria, a region where gender discrimination is prevalent.
• Data analysis can be carried out on the data to assess the impact of various socio-economic factors, such as marital status, level of education and income levels, on access to healthcare. This can help answer questions such as “does being well educated or wealthy improve the chances of receiving prompt services from health care professionals in rural areas?”
• The data can be used to train artificial intelligence tools, such as Natural Language Processing (NLP) and/or Named Entity Recognition (NER), to automatically identify prevalent medical symptoms in the region of study.
• Using advanced NLPs, ChatBots could be built to serve as triage solutions for first-line medical respondents.

## 2 Data Description

This article describes the datasets collected from respondents in northern Nigeria regarding equitable healthcare in the region. Data was collected in English and Hausa languages. The curated data files, saved in Microsoft Excel (XLSX) format, and associated questionnaire (in PDF format) are available in Ref. [2]. There are a total of 6 data files in the repository, the first MS Excel file contains responses from an initial pilot study. The second two and third files contain responses from the main study in English and Hausa languages respectively. The fourth MS Excel file contains English interpretations of Hausa words in the Hausa data file, while the fifth file is a codebook describing the variables in the other four data files. The sixth file is the questionnaire in PDF format.

**Fig. 1:**
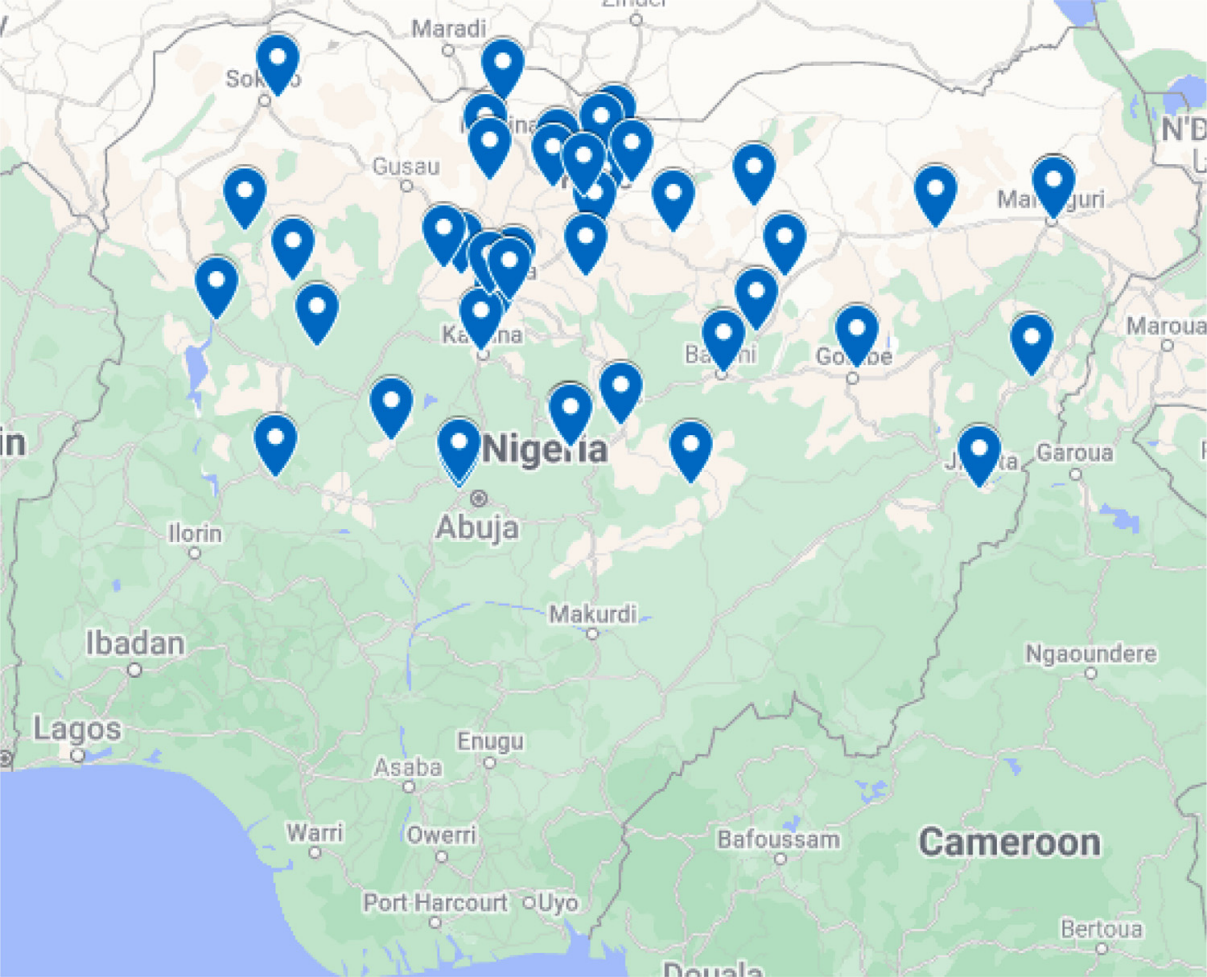
Map of Nigeria showing some of the locations where data were collected.

The collected data were from several towns and villages across the northern region of Nigeria as shown in Fig. 1. Data were collected using questionnaires administered using WhatsApp. The questionnaires had 43 questions, split into four sections, (i) demographic information, (ii) socio-economic information, (iii) wellness check, (iv) healthcare DEI; and a mix of three questions types, viz.: (i) open-ended questions, (ii) closed-ended questions with dropdown options (e.g., “yes/no”, “employed/unemployed/self-employed”), (iii) scaled questions (5 point likert scale). Table 1 provides a concise summary of the 43 questions types and corresponding variables in the datasets. 8 additional variables are included, named as ‘variableName_Translated’, which are related to the fourth dataset (English translation of the Hausa dataset). These additional variables are the English translations of their corresponding variables in Hausa language. Table 2 summarises the datasets and gives information of the collection period.

**Table 1:** Description of variables / fields in the datasets.

Question type	Field / Variable name	Options provided
A. Demographic information		
Open-ended questions	‘age’, ‘language’	Text field provided
Closed-ended questions	‘gender’, ‘askmarital’, ‘asklocation’, ‘askhavekids’, ‘howmanykids’, ‘howmanykids_Translated’, ‘nationality’, ‘ethnicity’.	Fixed options, e.g., Yes or No, Better or Worse
B. Social and economic information		
Open-ended questions	‘details’, ‘details_Translated’	Text field provided
Closed-ended questions	‘askeducation’, ‘askincome’, ‘askemployment’, ‘healthaccess’, ‘typeofhealthcare’, ‘socialsupport’, ‘askfamilysupport’	Fixed options, e.g., Yes or No, Better or Worse
C. Wellness check		
Open-ended questions	‘symptoms’, ‘symptoms_Translated’, ‘howlongsymptoms’, ‘howlongsymptoms_ Translated’, ‘howoftensmoke’, ‘medicinescurrent’, ‘medicinescurrent_Translated’, ‘medicinesrecent’, ‘medicinesrecent_Translated’, ‘describesurgeries’, ‘describesurgeries_Translated’	Text field provided
Closed-ended questions	‘wellness’, ‘gettingbetterorworse’, ‘medicines’, ‘familysymptoms’, ‘whichfamily’, ‘smoke’, ‘whichfamily_Translated’, ‘overallhealth’, ‘alcohol’, ‘howoftenalcohol’, ‘sex’, ‘partners’, ‘surgeries’.	Fixed options, e.g., Yes or No, Better or Worse
D. Healthcare - diversity, equity, & inclusion. [Likert scale from 1 to 5, with 1 = strongly disagree, 5 = strongly Agree]		
Scaled questions	‘inclusion’, ‘equity’, ‘diversity’, ‘genderjusticesafety’, ‘genderjusticecomfort’, ‘harassment’, ‘discrimination’, ‘refusedtreatment’	Likert scale 1 to 5

**Table 2:** Summary of datasets [2].

Dataset	Number of respondents	Number of variables	Collection period
Dataset 1 - “Gender_Equity_Northern_Nigeria_English_Responses_Phase_1”	1093	43	September 2022. Done as a Pilot study.
Dataset 2 - “Gender_Equity_Northern_Nigeria_English_Responses_Phase_2”	1606	43	January 2023. Main study.
Dataset 3 - “Gender_Equity_Northern_Nigeria_Hausa_Responses”	1929	43	March 2023. Main study.
Dataset 3b - “Gender_Equity_Northern_Nigeria_Hausa_Responses_with_Translations”	1929	51	March 2023. Main study.

Table 3 shows a summary of the distribution of the respondents per dataset and sheds light on the sample population considered.

**Table 3:** Distribution of sample population (respondents).

Variable	Dataset 1 (English)		Dataset 2 (English)		Dataset 3 (Hausa)	
	Class	%	Class	%	Class	%
Age	‘< 25 years’ - 530 ‘25–35’ - 422 ‘35–45’ - 104 ‘45–55’ - 28 ‘55–65’ - 8 ‘> 65 years’ - 2	48.5 38.6 9.5 2.6 0.7 0.2	‘< 25 years’ - 633 ‘25–35’ - 766 ‘35–45’ - 179 ‘45–55’ - 23 ‘55–65’ - 3 ‘> 65 years’ - 2	39.4 47.7 11.1 1.4 0.2 0.1	‘< 25 years’ -1273 ‘25–35’ - 599 ‘35–45’ - 46 ‘45–55’ - 6 ‘55–65’ - 2 ‘> 65 years’ - 3	66.0 31.1 2.4 0.3 0.1 0.2
Gender	Men - 383 Women - 695 Non-binary - 15	35.0 63.6 1.4	Men - 414 Women - 1182 Non-binary - 10	25.8 73.6 0.6	Men - 1134 Women - 727 Non-binary - 68	58.8 37.7 3.5
Location of Residence	Town - 947 Village - 136 NR - 10	86.6 12.4 0.9	Town - 1390 Village - 184 NR - 17	87.4 11.6 1.1	Town - 1239 Village - 640 NR- 40	64.6 33.4 2.1
Education Level	Primary - 13 Secondary - 336 Tertiary - 593 Others - 109 NR - 42	1.2 30.7 54.3 10.0 3.8	Primary - 17 Secondary - 575 Tertiary - 768 Others - 202 NR - 44	1.1 35.8 47.8 12.6 2.7	Primary - 105 Secondary - 1046 Tertiary - 646 Others - 109 NR - 101	5.2 52.1 32.2 5.4 5.0
Income Level	Level 1 - 22 Level 2 - 13 Level 3 - 11 Level 4 - 29 Level 5 - 234 Level 6 - 726 NR - 58	2.0 1.2 1.0 2.7 21.4 66.4 5.3	Level 1 - 36 Level 2 - 20 Level 3 - 24 Level 4 - 43 Level 5 - 295 Level 6 - 1126 NR- 62	2.2 1.2 1.5 2.7 18.4 70.1 3.9	Level 1 - 169 Level 2 - 71 Level 3 - 53 Level 4 - 96 Level 5 - 395 Level 6 - 1020 NR- 125	8.8 3.7 2.7 5.0 20.5 52.9 6.5
Employment	Employed - 283 Self-employed-433 Unemployed - 318 NR - 59	25.9 39.6 29.1 5.4	Employed - 450 Self-employed-755 Unemployed - 333 NR - 68	28.0 47.0 20.7 4.2	Employed - 481 Self-employed-529 Unemployed - 790 NR - 129	24.9 27.4 41.0 6.7

NR= No Response.

Regarding the response rates, of the number of respondents who participated in the data collection process, 21% completed the survey and answered all questions, while 53% provided partial responses. Partial responses means that certain questions were left unanswered or skipped. Finally, data collection was a one-off process for each respondent, with no deadlines set or reminders sent to respondents.

Though the data provided here are intended as inputs or precursors to more detailed analyses, some quick insights can be drawn from them. For instance, from the distribution of respondents in the data, as shown on Table 3, it can be seen that despite the widespread stereotyping and repression of women in northern Nigeria, more women participated in the survey than any other genders. This perhaps suggests that providing a safe space and an enabling environment might be instrumental in tackling the repression against women. Further, the distribution of the respondents also reveals that a large percentage of the population are youths aged 35 years and younger, who live in Towns and have at least secondary school level education. Despite this youthful population, the high level of insecurity and constant insurrections in the region are perhaps responsible for limiting the economic power of residents, as most earn less than USD 500 annually.

## 3 Experimental Design, Materials and Methods

### 3.1 Questionnaire Structure

• The data were collected using an anonymously administered questionnaire.
• The questions were designed to be simple and unambiguous, such as ‘How old are you (years)?’ or ‘What Nigerian ethnic group do you belong to?’
• The questionnaire had three question types - open-ended, closed-ended, and 5 point likert scale questions. 10 of the 43 questions were open-ended, for which text fields were provided into which the respondents could type in their responses. One such question was regarding the symptoms being experienced by the respondents. Responses provided here could be passed into artificial intelligence tools for automatic symptom elicitation and inferences.  25 of the 43 questions were closed-ended questions, with respondents provided dropdown options to choose from. For instance, a question such as ‘What is your marital status?’, had four possible response options (‘Single’, ‘Married’, ‘Divorced’, ‘Widow/Widower’) from which the respondent could choose. The remaining 8 questions were 5 point likert scale, ranging from 1 (strongly disagree) to 5 (strongly agree).

### 3.2 Data Collection Method

• All data were collected *via* askNivi, a conversational health tool accessible *via* WhatsApp. The primary purpose of askNivi is to provide health education and referrals for healthcare systems [1].
• Participants (respondents) were patients or visitors to healthcare centres in villages and towns in northern Nigeria.
• Respondents were offered incentives to complete the questionnaire in the form of mobile airtime credit (as most mobile lines are prepaid in Nigeria). During the pilot study, the 1093 respondents were split into 3 groups of 364, 364, and 365, respectively. They were then offered NGN 500, NGN 1500, and NGN 2500 (USD 0.50, USD 1.50, USD 2.50) as incentives to fill the questionnaire. However, no significant difference in response rate was observed across the three groups. At NGN 500, only 84 of the 364 participants (23%) responded; at NGN 1500 there were only 77 (21%) responses, and only 70 (19%) responded at NGN 2500. Based on this, respondents were offered an incentive of NGN 500 during the main data collection phase.
• During the main data collection phase, an askNivi link was sent to the respondents mobile phones *via* whatsapp. Literate respondents filled out the questionnaires by themselves while non-literate respondents (who chose to participate), were assisted by literate family members or administrative staff of the healthcare centres.
• Data were collected between September 2022 and March 2023.

## Limitations

• In northern Nigeria, women are often not allowed to go out on their own or interact with the general public without a male chaperone (in the person of her father, husband, or brother). This severely limits womens' freedom of expression and ability to provide unbiased / uninfluenced responses to the questions.
• Non-binary gender is not legally recognized in Nigeria, hence the small number of respondents who identified as non-binary.
• In the region, men are often not as open to discussing their personal / medical conditions as women, hence why there were more female respondents than other genders.
• During the pilot study, the data collection process was not well moderated hence there were lots of incorrect or invalid responses in the data.
• For the open-ended questions, there were several typographical errors in the provided responses which made automatic preprocessing a challenge. To use the data for further analysis, significant manual preprocessing would be required. For example, omission of spaces between words or misspelt words, especially those related to the medical symptoms, can be challenging.

## Ethics Statement

1. Data collected were from respondents who gave their consent to participate. All respondents were presented with a first page on the Nivi app, which clearly stated that the data being collected was for research and analytic purposes, and would remain completely anonymous without any form of tracking. All respondents were required to accept these terms and conditions before being allowed to participate in the data collection exercise. A copy of this consent form has been submitted with this article.
2. This study is institutional review board exempt, as the data collection process relied exclusively on surveys and data was collected anonymously, such that the identity of the respondents cannot be ascertained [3].
3. Data were collected using askNivi, an app specifically designed for healthcare related data collection. Privacy policy, consent, and related information about askNivi can be found at www.nivi.io/privacy-policy
4. In the region of interest, northern Nigeria, almost 50 % of girls are married and are parents to multiple kids by the age of 15 [4,5]. This is also reflected in our collected data. For this study, the investigators ensured that all respondents were at least 15 years old.

## CRediT authorship contribution statement

**Chika Yinka-Banjo:** Conceptualization, Methodology, Supervision. **Mary Akinyemi:** Validation, Methodology, Writing – review & editing. **Olasupo Ajayi:** Investigation, Software, Writing – original draft. **David Tresner-Kirsch:** Data curation, Software, Methodology.

## Data Availability

Data on Gender-Equitable Healthcare Accessibility in Northern Nigeria (Original data) (Mendeley Data).
